# A splenic artery aneurysm presenting with multiple episodes of upper gastrointestinal bleeding: a case report

**DOI:** 10.1186/s13256-017-1282-7

**Published:** 2017-05-03

**Authors:** W. S. L. De Silva, D. S. Gamlaksha, D. P. Jayasekara, S. D. Rajamanthri

**Affiliations:** 1 Post Graduate Institute of Medicine, University of Colombo, Colombo, Sri Lanka; 2 Vascular and Transplant Surgical Unit, National Hospital of Sri Lanka, Colombo, Sri Lanka

**Keywords:** Splenic artery aneurysm, True aneurysm, Double rupture, Intragastric bleeding, Case report

## Abstract

**Background:**

Splenic artery aneurysm is rare and its diagnosis is challenging due to the nonspecific nature of the clinical presentation. We report a case of a splenic artery aneurysm in which the patient presented with chronic dyspepsia and multiple episodes of minor intragastric bleeding.

**Case presentation:**

A 60-year-old, previously healthy Sri Lankan man presented with four episodes of hematemesis and severe dyspeptic symptoms over a period of 6 months. The results of two initial upper gastrointestinal endoscopies and an abdominal ultrasound scan were unremarkable. A third upper gastrointestinal endoscopy detected a pulsatile bulge at the posterior wall of the gastric antrum. A contrast-enhanced computed tomogram of his abdomen detected a splenic artery aneurysm measuring 3 × 3 × 2.5 cm. While awaiting routine surgery, he developed a torrential upper gastrointestinal bleeding and shock, leading to emergency laparotomy. Splenectomy and *en bloc* resection of the aneurysm with the posterior stomach wall were performed. Histology revealed evidence for a true aneurysm without overt, acute, or chronic inflammation of the surrounding gastric mucosa. He became completely asymptomatic 2 weeks after the surgery.

**Conclusions:**

Splenic artery aneurysms can result in recurrent upper gastrointestinal bleeding. The possibility of impending catastrophic bleeding should be remembered when managing patients with splenic artery aneurysms after a minor bleeding. Negative endoscopy and ultrasonography should require contrast-enhanced computed tomography to look for the cause of recurrent upper gastrointestinal bleeding.

## Background

Splenic artery aneurysm (SAA) is rare, although it is considered to be the third-most common site for intra-abdominal aneurysms and the most common for splanchnic aneurysms [1]. Incidence ranges from 0.09% in autopsy studies to 0.78% on arteriography studies [2]. Pseudoaneurysms of the splenic artery are rarer, but take bigger sizes and more catastrophic courses than true aneurysms. SAAs are mostly found incidentally, in imaging studies done for various non-related symptoms [3]. Ruptured SAAs can present as acute abdomen with intraperitoneal bleeding, essentially making it a retrospective diagnosis, which often has grave outcomes [4]. Of interest, the rupture of a SAA is more common in males, although SAAs are four times more common in females than in males [3]. We report a case of a 60-year-old man presenting chronic dyspeptic symptoms and several episodes of minor upper gastrointestinal bleeding due to an SAA. The diagnosis of the condition was delayed for 6 months due to non-compliance for advanced imaging, the repeatedly normal endoscopic appearance of the patient’s upper gastrointestinal tract, and the remarkably stable clinical course of the patient in-between minor bleeds, until an episode of torrential intragastric bleeding resulting in hemorrhagic shock.

## Case presentation

**Fig. 1:**
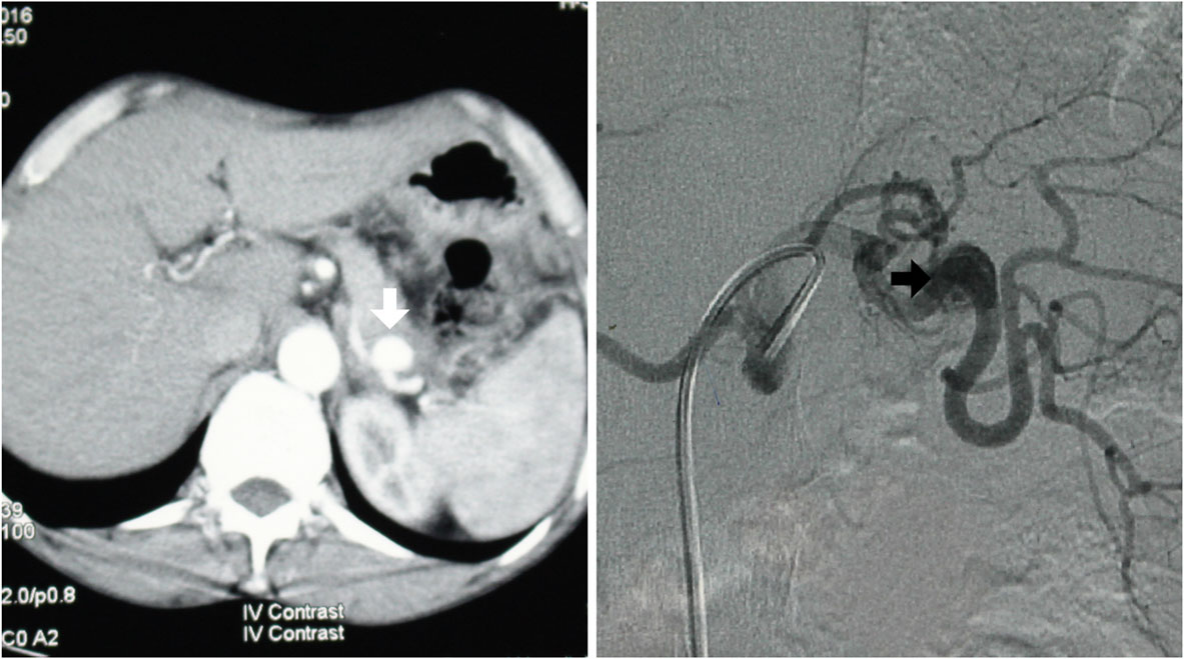
Imaging of the splenic artery aneurysm. *Left* panel shows contrast-enhanced computed tomography film of the abdomen illustrating the splenic artery aneurysm (*white arrow*). *Right* panel shows celiac axis digital subtraction assay showing the splenic artery aneurysm involving the middle third of the splenic artery (*black arrow*)

A 60-year-old, previously healthy Sri Lankan man was referred to the National Hospital of Sri Lanka, Colombo, for further evaluation of recurrent hematemesis for 6 months. Prior to this, he had four episodes of hematemesis which were all self-limiting. He had severe dyspeptic symptoms for the same duration, which was managed with antacids by his general practitioner. He denied any symptom improvement for antacids. He was admitted to a primary care hospital during the third and fourth episodes of hematemesis, where he was initially investigated. Two upper gastrointestinal endoscopies had only revealed evidence of mild erosive gastropathy at the antrum. An ultrasound scan of his abdomen was also normal. His transfer to a tertiary care hospital for further investigation was delayed for 2 months due to poor compliance. He was not anemic and the rest of his physical examination was unremarkable. A third upper gastrointestinal endoscopy was done at the tertiary care hospital because he continued to have severe dyspeptic symptoms. It detected a pulsatile mass at the lesser curvature of the gastric antrum in an otherwise normal study. Based on this information, a contrast-enhanced computed tomography of his abdomen was done, which showed a 3 × 3 × 2.5 cm SAA in the middle third of the artery, in relation to the lesser curvature of his stomach. This finding was further confirmed in a celiac axis digital subtraction assay (Fig. 1).

**Fig. 2:**
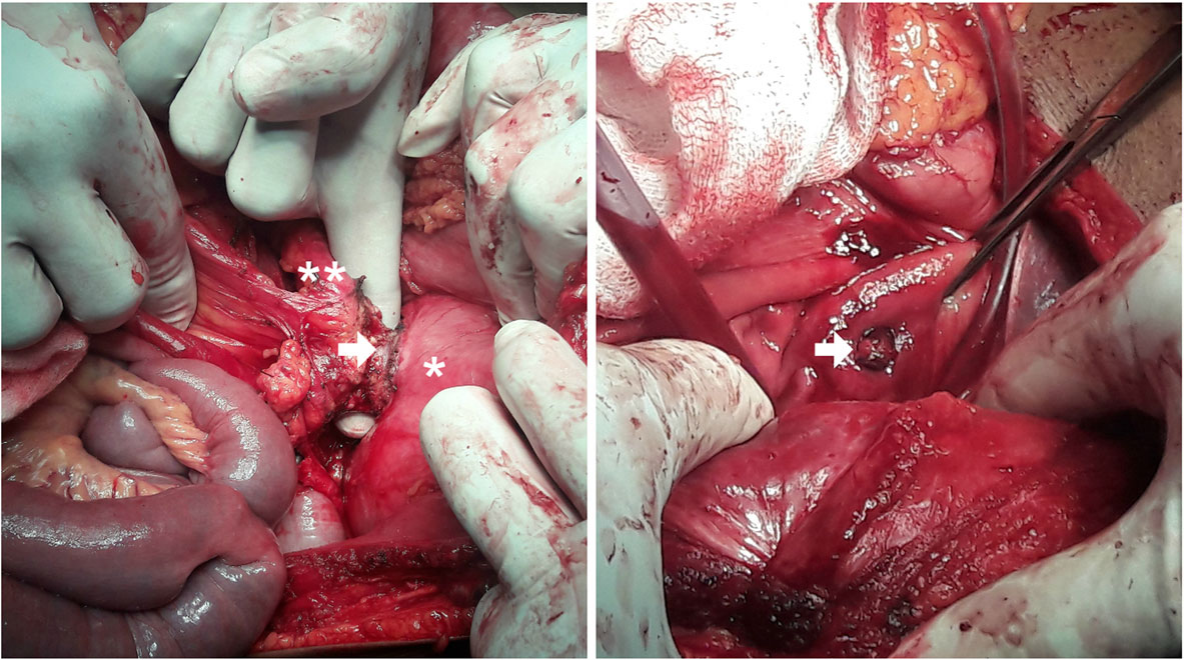
Intraoperative findings. *Left* panel shows aneurysm wall (*arrow*) densely adhered to the posterior wall of the stomach (*), macroscopically normal pancreas after mobilization (**). *Right* panel shows interior of the stomach showing the fistula occluded by a thrombus arising from the aneurysm sac (*arrow*)

He was prepared for open surgery in 2 weeks, and was given vaccines to prepare for splenectomy. Coil embolization of the aneurysm was not planned due to the non-availability of suitable size coils. One day prior to the scheduled date for surgery, he developed massive hematemesis, resulting in hemorrhagic shock. An emergency exploration was performed with concurrent resuscitation, with a massive blood transfusion. His stomach was filled with approximately 3 L of blood, necessitating anterior gastrotomy to decompress his stomach prior to exploration. The lesser sac was opened and the origin of his splenic artery was clamped. The aneurysm was found to erode into his stomach, at the lesser curvature of the antrum with dense induration (Fig. 2). His splenic artery was ligated proximal to the aneurysm, and splenectomy was performed. An ulcer measuring 0.8 cm at the posterior wall of the antrum was noted communicating with the aneurysm sac (Fig. 2). The ulcer was excised with a cuff of normal stomach tissue en bloc with the aneurysm.

**Fig. 3:**
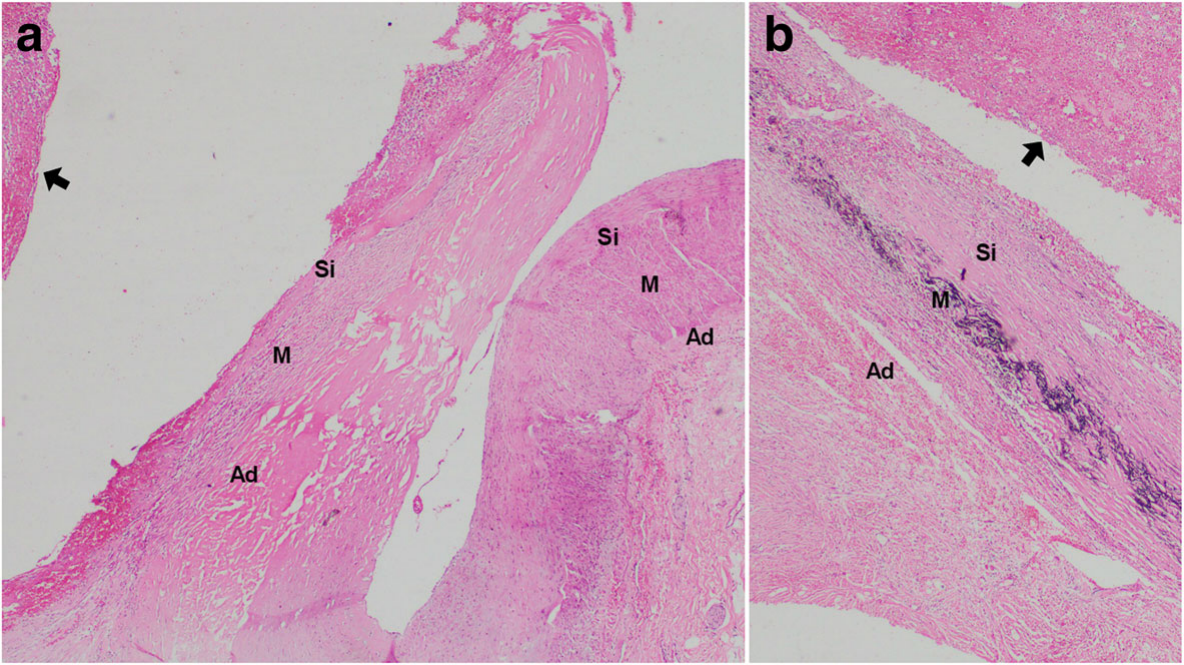
Histology of the aneurysm. The wall of the artery showing all three components of the vessel wall: subintimal layer, media, and the adventitia. Intima is not clearly identified. The hematoma is seen outside the subintimal layer (*arrow*) (**a**). Focal areas of dystrophic calcification due to degeneration of media were identified (basophilic strip in the media of slide – (**b**). *Ad* adventitia, *M* media, *Si* subintimal layer

His postoperative period was uneventful, and he was discharged on the tenth postoperative day. Histology of the resected specimen showed a true aneurysmal sac eroding into minimally inflamed gastric mucosa at the site of the mucosal breach. The surrounding gastric mucosa did not have evidence of acute or chronic inflammation. The degeneration of the media of the aneurysm wall manifested itself as dystrophic calcification (Fig. 3). Our patient became completely asymptomatic with regard to chronic dyspepsia, following surgery.

## Discussion

SAAs are mostly detected incidentally in emergency exploratory laparotomies done for hemoperitoneum, apart from the incidentally found SAAs in various imaging studies. Upper abdominal pain is the most commonly reported symptom for ruptured SAAs [5, 6]. The clinical presentation of unruptured SAA is largely nonspecific and variable [7, 8]. Our patient had severe dyspeptic symptoms for 6 months with minimal abnormalities detected in endoscopic evaluation of his upper gastrointestinal tract. The complete resolution of the symptoms after the excision of the aneurysm and the repeatedly normal endoscopic appearance of gastric mucosa suggest that his chronic dyspeptic symptoms were due to the aneurysm itself. It is not uncommon to observe gastric symptoms resulting from large pseudoaneurysms of the splenic artery. Of interest, in this case, it was a small, spontaneous, true SAA that gave rise to the symptoms. Thus, a mechanism other than pure physical compression of the stomach has to be suspected, resulting in dyspeptic symptoms.

Rupture risk is very low (2 to 3%) for true aneurysms, but it is alarmingly high for pseudoaneurysms (37 to 47%) with 90% mortality [9]. A spontaneous rupture of true SAAs is encountered more with aneurysms larger than 2 cm in diameter and with aneurysms in pregnant women [10–14]. Patients with intraperitoneal rupture of a SAA present with acute abdomen and hypovolemic shock [15, 16]. Bleeding into the stomach is rare with true SAAs. A few cases of possibly true SAAs with intragastric bleeding were reported, but the histologic confirmation of them being true aneurysms was not confirmed [17, 18]. Unlike true SAAs, intragastric bleeding is a common feature of pseudoaneurysms of the splenic artery [19, 20]. A rare case of a splenic artery pseudoaneurysm fistulating into the transverse colon was reported by O’Brien *et al.* [21].

“Double rupture” is a recognized phenomenon for intraperitoneal bleeding of true SAA, with an initial, brief, arrested bleeding into the lesser sac followed by massive bleeding into the peritoneal cavity. Recurrent intragastric bleeding with a stable course over a long period was seen in our patient. The initial endoscopic evaluations were unable to detect any significant abnormality, and the diagnosis was only unveiled with computed tomography of his abdomen. The long delay in making the diagnosis was largely due to the late presentation to a primary care hospital and to the non-compliance of the patient in getting transferred to a tertiary care hospital for advanced imaging. Detecting a pulsatile bulge at the posterior stomach wall expedited arranging contrast-enhanced computed tomography, in this case. The fortunate occurrence of having the first torrential upper gastrointestinal bleeding in a hospital setup with vascular surgical capacities helped to save the life of the patient.

A few cases were reported with interesting workup, to diagnose SAA. A similar case of double rupture of a splenic artery pseudoaneurysm, with initial negative endoscopic and ultrasonography evidence, was reported by Sawicki *et al.* [19]. Another case of a SAA, first suspected after seeing a non-pulsatile gastric lesion during endoscopy, was reported by Tannoury *et al.* [22]. Boschmann *et al.* reported a case where the ultrasound scan of the abdomen was useful in suspecting a SAA in a patient with recurrent gastrointestinal bleeding [23].

No universally accepted guidelines are available for the management of SAA. However, a number of case series and reviews have outlined a few principles for patient management. Most small (<2.0 cm) asymptomatic SAAs can be monitored effectively with serial imaging [9]. The advent of endovascular techniques to embolize aneurysms has gained popularity over the last decade due to the low morbidity. Transcatheter embolization can be performed with gelatin gels, steel coils, detachable balloons, or glue material. Yet, open surgical exploration and aneurysmectomy remain the gold standard in the management of SAA [1]. Open surgery is essentially the only resort in cases with giant SAA, ruptured SAA, and with SAA complicated with other local and regional pathologies.

## Conclusions

The diagnosis of SAA should be considered when no other common pathologies are identified for recurrent upper gastrointestinal bleeding. Endoscopy and ultrasonography are not helpful in excluding an SAA. The possibility of double or multiple ruptures should be borne in mind when managing patients with SAA, after falsely showing hemodynamic stability. Although rare, true SAAs also can result in intragastric rupture with catastrophic gastrointestinal bleeding.

## Abbreviation

SAA: Splenic artery aneurysm
